# Tractography-assisted deep brain stimulation of the superolateral branch of the medial forebrain bundle (slMFB DBS) in major depression

**DOI:** 10.1016/j.nicl.2018.08.020

**Published:** 2018-08-14

**Authors:** Volker A. Coenen, Bastian Sajonz, Marco Reisert, Jan Bostroem, Bettina Bewernick, Horst Urbach, Carolin Jenkner, Peter C. Reinacher, Thomas E. Schlaepfer, Burkhard Mädler

**Affiliations:** a Department of Stereotactic and Functional Neurosurgery, Freiburg University Medical Center, Germany; b Division of Interventional Biological Psychiatry, Department of Psychiatry and Psychotherapy, Freiburg University Medical Center, Germany; c Department of Neuroradiology, Freiburg University Medical Center, Germany; d Medical Faculty, Freiburg University, Freiburg, Germany; e Department of Neurosurgery, Bonn University Medical Center, Germany; f Department of Psychiatry and Psychotherapy, Geriatric Psychiatry and Neurodegenerative Disorders, Bonn University Medical Center, Germany; g Division of Neuroradiology, Department of Radiology, Bonn University Medical Center, Germany; h BrainLinks/BrainTools, Cluster of Excellence, Freiburg University, Germany; i Clinical Trials Unit, Freiburg University, Germany; j Philips GmbH DACH, Hamburg, Germany

**Keywords:** Deep brain stimulation, Depression, Diffusion tensor imaging, Fiber tracking, Medial forebrain bundle, OCD, slMFB, Stereotactic surgery, Tractography, CT, computed tomography, DBS, deep brain stimulation, DTI, diffusion tensor magnetic resonance imaging, DTI FT, DTI fiber tractography, EC, effective contact, FT, fiber tractography, HF, high frequency, Hz, Hertz [1/s], IPG, internal pulse generator, mA, milli-ampere, MADRS, Montgomery-Åsberg Depression Rating Scale, MCP, mid-commissural point, MDD, major depressive disorder, MRI, magnetic resonance imaging, RN, red nucleus, STN, subthalamic nucleus, SNr, substantia nigra pars reticulata, VAT, volume of activated tissue, VTA, ventral tegmental area, μs, micro second

## Abstract

**Background:**

Deep brain stimulation (DBS) of the superolateral branch of the medial forebrain bundle (slMFB) emerges as a - yet experimental - treatment for major depressive disorder (MDD) and other treatment refractory psychiatric diseases. First experiences have been reported from two open label pilot trials in major depression (MDD) and long-term effectiveness for MDD (50 months) has been reported.

**Objective:**

To give a detailed description of the surgical technique for DBS of the superolateral branch of the medial forebrain bundle (slMFB) in MDD.

**Methods:**

Surgical experience from bilateral implantation procedures in *n* = 24 patients with MDD is reported. The detailed procedure of tractography-assisted targeting together with detailed electrophysiology in 144 trajectories in the target region (recording and stimulation) is described. Achieved electrode positions were evaluated based on postoperative helical CT and fused to preoperative high resolution anatomical magnetic resonance imaging (MRI; Philips Medical Systems, Best, Netherlands), including the pre-operative diffusion tensor imaging (DTI) tractographic information (StealthViz DTI, Medtronic, USA; Framelink 5.0, Medtronic, USA). Midcommissural point (MCP) coordinates of effective contact (EC) location, together with angles of entry into the target region were evaluated. To investigate incidental stimulation of surrounding nuclei (subthalamic nucleus, STN; substantia nigra, SNr; and red nucleus, RN) as a possible mechanism, a therapeutic triangle (TT) was defined, located between these structures (based on MRI criteria in T2) and evaluated with respect to EC locations.

**Results:**

Bilateral slMFB DBS was performed in all patients. We identified an electrophysiological environment (defined by autonomic reaction, passive microelectrode recording, acute effects and oculomotor effects) that helps to identify the proper target site on the operation table. Postoperative MCP-evaluation of effective contacts (EC) shows a significant variability with respect to localization. Evaluation of the TT shows that responders will typically have their active contacts inside the triangle and that surrounding nuclei (STN, SNr, RN) are not directly hit by EC, indicating a predominant white matter stimulation. The individual EC position within the triangle cannot be predicted and is based on individual slMFB (tractography) geometry. There was one intracranial bleeding (FORESEE I study) during a first implantation attempt in a patient who later received full bilateral implantation. Typical oculomotor side effects are idiosyncratic for the target region and at inferior contacts.

**Conclusion:**

The detailed surgical procedure of slMFB DBS implantation has not been described before. The slMFB emerges as an interesting region for the treatment of major depression (and other psychiatric diseases) with DBS. So far it has only been successfully researched in open label clinical case series and in 15 patients published. Stimulation probably achieves its effect through direct white-matter modulation of slMFB fibers. The surgical implantation comprises a standardized protocol combining tractographic imaging based on DTI, targeting and electrophysiological evaluation of the target region. To this end, slMFB DBS surgery is in technical aspects comparable to typical movement disorder surgery. In our view, slMFB DBS should only be performed under tractographic assistance.

## Highlights

• The slMFB is an emerging target for DBS in therapy refractory Depression.
• The therapeutic effect is related to modulation of white matter.
• Surgery for slMFB DBS is tractography assisted surgery.
• DBS of the slMFB is in many aspects similar to movement disorder surgery.

## Graphical abstract

**Figure:**
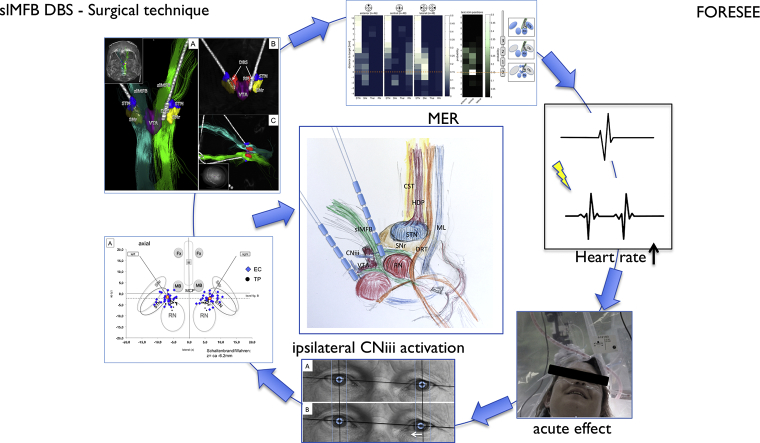

## 1 Introduction

Major depression is a prevalent disorder and according to the World Health Organization (http://www.who.int/news-room/fact-sheets/detail/depression, assessed 3 June 2018) >300 million people are affected worldwide. Despite effective therapies, 20% of patients will ultimately remain treatment resistant (Schlaepfer et al., 2014). Deep brain stimulation for the treatment of major depressive disorder (MDD) is a rather new indication offering hope for some of these treatment resistant patients. A first uncontrolled case series studied effects of DBS to Brodman area 25 (cg25, later termed SCG = subgenual cingulate gyrus) (Mayberg et al., 2005) and the scg target is likely the most frequently implanted structure in this disease (Kisely et al., 2018). Other target regions have been researched (ALIC = anterior limb of the internal capsule; vc/vs = ventral capsule ventral striatum for which case series exist and some smaller case series or even single case reports for structures like inferior thalamic peduncle, habenula and others). For a review on the topic refer to (Hariz et al., 2013; Kisely et al., 2018). Two pivotal randomized controlled multicentric trials were recently stopped after futility analysis (Dougherty et al., 2015; Holtzheimer et al., 2017). Despite these set-backs there is interest in the psychiatric and neurosurgical communities to explore DBS in this indication. One of the latest additions to tentative target regions (Schlaepfer et al., 2013) is the superolateral branch of the medial forebrain bundle (slMFB) a structure that is involved in both reward anticipation and reward perception in vertebrates. Structurally it is confluent with the mesolimbic dopaminergic system although many other neurotransmitters have been found to be associated with its function (Coenen et al., 2009; Coenen et al., 2011; Coenen et al., 2012; Coenen et al., 2018). We presented comprehensive reasoning as to why to select the slMFB as a target region in MDD previously (Coenen et al., 2011; Schlaepfer et al., 2014). So far, human slMFB anatomy was described purely based on the diffusion tensor magnetic resonance imaging (DTI) technology. After the first anatomical description and the theory about antidepressant efficacy of slMFB stimulation it became clear that the implantation had to be informed by tractography. First results of our own and another group's open label trials on short- and long-term efficacy are promising (Bewernick et al., 2017; Fenoy et al., 2016; Schlaepfer et al., 2013). The slMFB has been the first target for DBS truly defined by tractography; this targeting technology has now been adapted for yet another target region (scg) for MDD in order to increase therapeutic efficacy in a region that is silent with respect to electrophysiology and other biomarkers during implantation (Riva-Posse et al., 2014; Riva-Posse et al., 2017).

This article aims at sharing our detailed neurosurgical experience of slMFB DBS in *n* = 24 bilateral implantation procedures from two open label clinical trials (FORESEE I & FORESEE II) in MDD. Our goal is to encourage other groups to research this target region.

## 2 Material and methods

We report our experience in 24 cases of bilateral slMFB DBS in MDD who were implanted during two open label trials (FORESEE & FORESEE II).

### 2.1 Ethics

Both trials were conducted under the tenets of the Declaration of Helsinki. All patients gave written informed consent for participation in the studies. Both trials were reviewed and positively evaluated by the Bonn University Medical Center ethics committee.

### 2.2 Imaging

MR imaging data were acquired on a whole-body 3 T MR system (Philips Healthcare, Best, The Netherlands) by using an 8-element phased-array head coil. The MR imaging examination comprised an isotropic T2-weighted fast spin-echo sequence, a DTI sequence, and 2 magnetization-prepared rapid gradient- echo scans. The parameters were the following: fast spin-echo: repetition time (TR) = 12.650 ms, echo time (TE) = 100 ms, field of view (FOV) = 254 mm, matrix = 176 · 176, 120 sections, sections thickness = 1.44 mm, and acquisition time = 3 min and 44 s. The resulting data were reconstructed to isotropic (1.44 · 1.44 · 1.44)-mm3 voxels.

Diffusion Tensor Imaging sequence: Single-shot spin-echo echo planar imaging pulse sequence with TR = 13.188 ms, TE = 84 ms,FOV = 256 mm,matrix = 128·128, 70 sections, section thickness = 2 mm, number of gradient directions = 32, b-value = 1000 s/mm2, sensitivity encoding factor 2.9, acquisition time = 7 min 54 s with isotropic reconstructed (2 · 2 · 2) mm3 voxels. A T1-weighted 3-D magnetization-prepared rapid gradient-echo sequence was acquired before (structural information) and after (vessel visualization) contrast administration (gadolinium-diethylene-triamine pentaacetic acid) with a sensitivity encoding factor = 4, TR = 8.5 ms, TE = 3.8 ms, flip angle = 8, FOV = 256 mm, matrix = 256 · 256, 160 sections, section thickness = 2 mm, acquisition time = 4 min 17 s. It resulted in reconstructed isotropic (1 · 1 · 1) mm3 voxels. All images were obtained in axial orientation.

*Preoperative* stereotactic computed tomography (CT) scans were acquired on a 16-row multidetector scanner (Brilliance 8000, Philips Healthcare) with a head mounted stereotactic frame. Parameters were as follows: tube voltage = 120 kV, tube current = 350 mA, collimation = 16 · 0.75 mm, tube rotation time = 1 s, pitch = 0.942, matrix = 512 · 512, section thickness = 1.5 mm, increment = 1.5 mm.

*Postoperative* helical CT used the following parameters: tube voltage = 120 kV, tube current = 350 mA, collimation = 16 · 0.75 mm, tube rotation time = 0.75 s, pitch = 0.688, matrix = 512 · 512, section thickness = 2 mm, increment = 1 mm.

### 2.3 Fiber tracking

Deterministic FT was performed on a Linux workstation using StealthViz DTI (Medtronic Navigation, Louisville, Colorado). After Eddy-current correction for bulk motion of the patient between scans, the B0 sequence was co-registered to the T2W high resolution anatomical imaging.

**Fig. 1:**
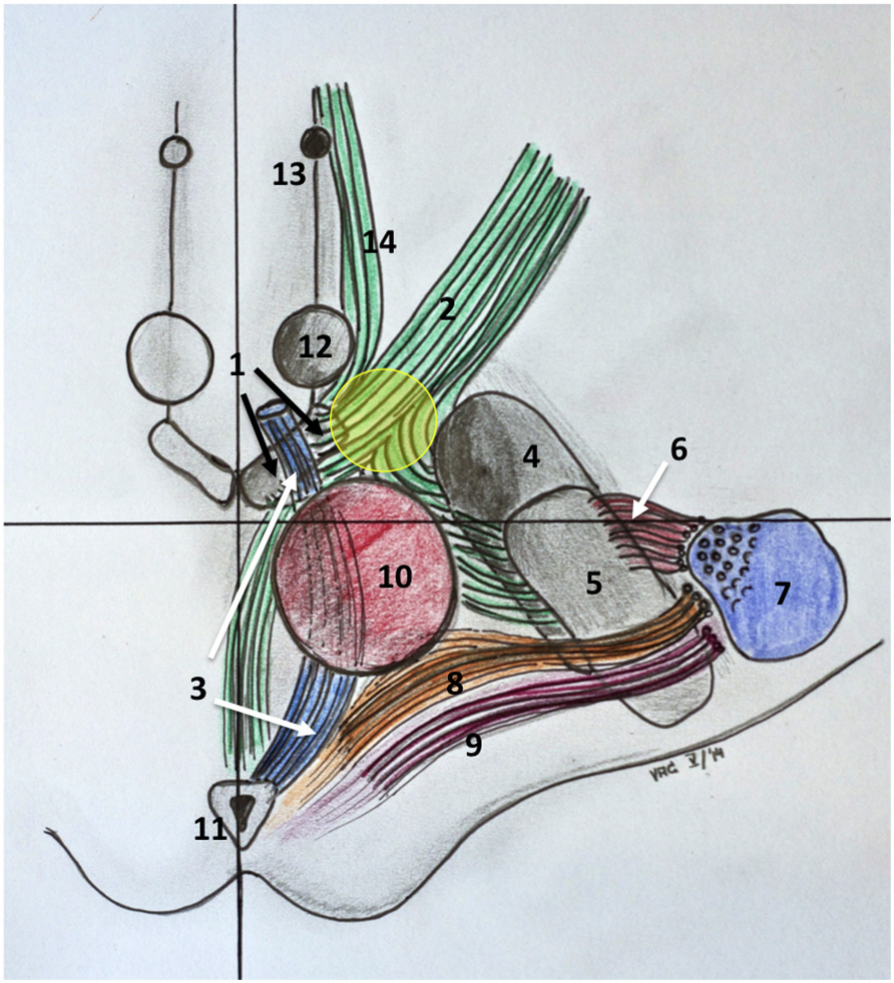
Artistic representation of the slMFB and the stimulated region. The stimulated region is located (yellow sphere) between the mammillary-bodies, the red nucleus and the anterior most aspect of the subthalamic nucleus. Note the proximity of the target region and the occulomotor nerve that traverses the VTA, laterally. Structures: 1, Ventra tegmental area (black arrows); 2, superolateral branch of medial forebrain bundle; 3, occulomotor nerve (CNiii, white arrows); 4, substantia nigra; 5, subthalamic nucleus; 6, hyperdirect pathway; 7, corticospinal tract; 8, dentato-rubro-thalamic tract; 9, medial lemniscus; 10, red nucleus; 11, periaquaeductal grey; 12, mammillary body; 13, fornix; 14, inferomedial branch of the medial forebrain bundle.

**Fig. 2:**
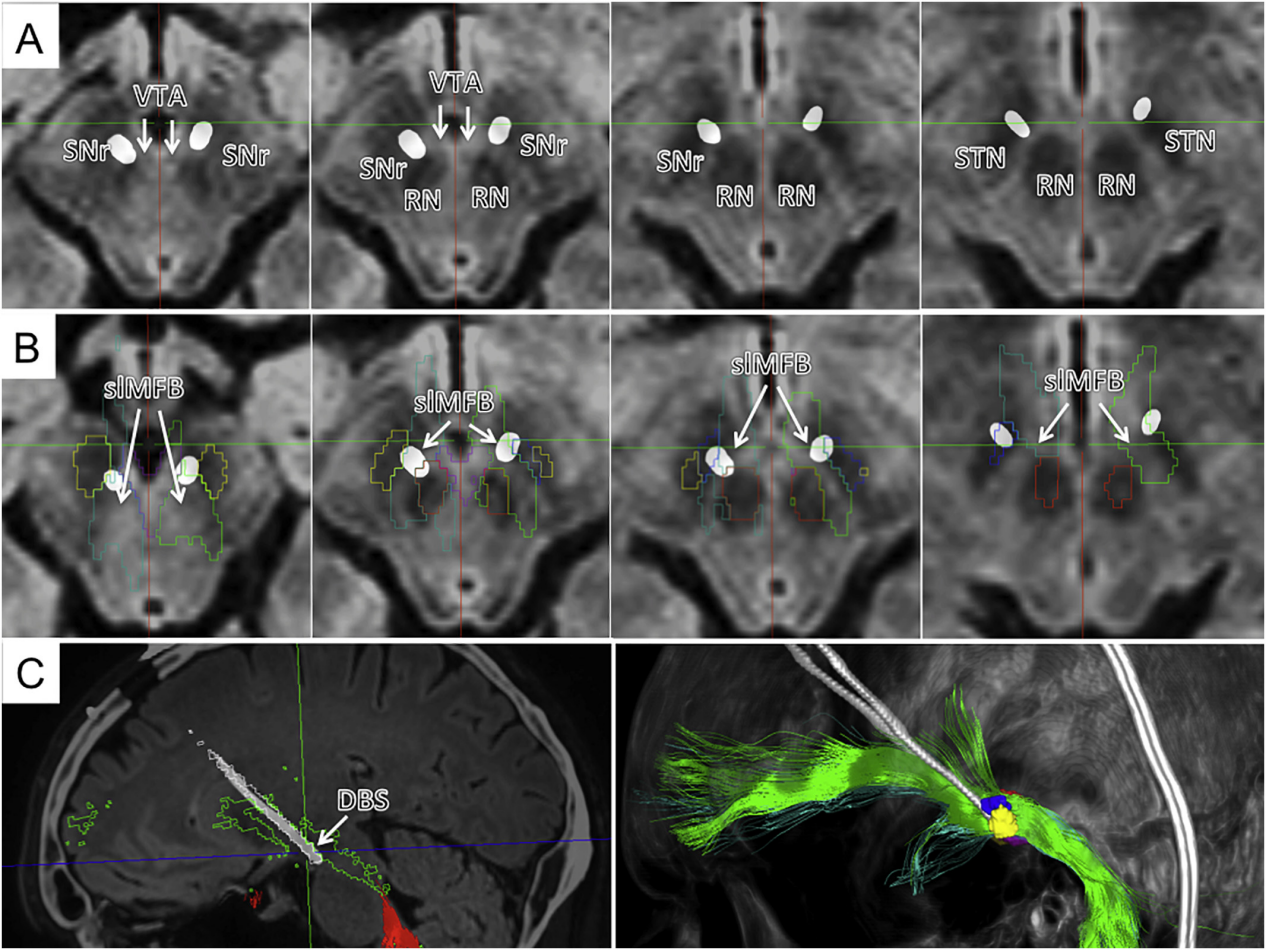
Typical slMFB DBS. A, axial slides showing deepest (left) and most superficial contacts (right) on T2-weighted anatomy. B, Outlines of functional structures given. C; left, outline shows how DBS electrode traverses the slMFB (green); right, three-dimensional view from lateral and left.

**Fig. 3:**
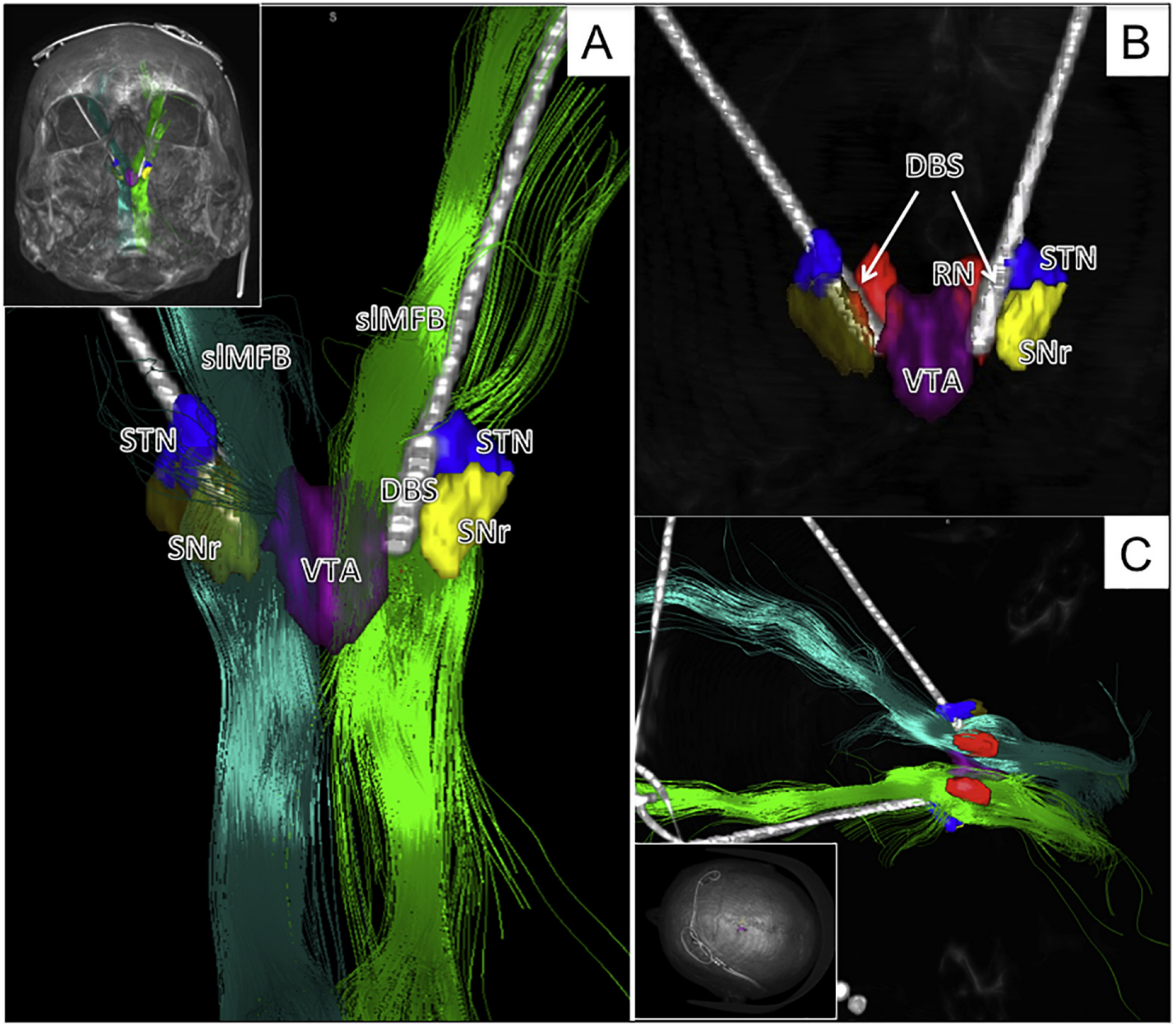
Three-dimensional depiction of a typical bilateral slMFB-DBS implantation. A, implantation site as viewed from sub-mentally. The DBS electrodes are situated inside the slMFB (green bundles) in the corridor medial to the STN/SNr-complex. The tip of the electrode touches the ventral tegmental area (VTA). B, same as A but without fibers. C, view from superior and left.

Fractional anisotropy was set at 0.2. Seed density was held at 5.0. Minimal fiber length was set to 20 mm. The MFB was tracked using a single rectangular region of interest (VOI box, 5x5x5 mm^3) which was placed in the white matter just lateral to the ventral tegmental area (VTA), as identified in the T2W high-resolution MRI (cf. Fig. 2). Essentially, the therapeutic triangle (TT, cf. Fig. 1, Fig. 2, Fig. 3) in an axial plane showing the widest red nucleus cross-sectional diameter was chosen. The anterior border of our VOI box was the ipsilateral mammillary body and the mammillothalamic tract. Laterally, the VOI box extended to the medial border of the subthalamic nucleus/substantia nigra complex (STN/SNr). The VOI box as tilted and deformed in order to best fit the individual anatomical situation (white matter in the TT), using the correlated tri-planar display. The detailed procedure has also been described before (Coenen et al., 2009; Coenen et al., 2012; Schlaepfer et al., 2013).

### 2.4 Planning

Bilateral and pre-coronal (coronal) entry points were chosen. At first a trajectory was defined that entered the center of the TT based on T2W MRI information. The tip of the electrode was then defined as just reaching the VTA (according T2W MRI). The trajectory was then adjusted in order to penetrate the center of the string-like structure of the slMFB (cf. Fig. 3). The definitive planned depth of implantation was determined agin with the overlayed tractographic slMFB rendition. The deepest point was defined as the inferior most limit of the slMFB. T1W imaging with contrast enhancement was used to exclude vessels collisions and allow a 2–3 mm safety margin. The trajectory was adjusted, accordingly.

### 2.5 Surgical procedure

After administration of standard antibiotic prophylaxis, a stereotactic frame (Leksell, Elekta, Stockholm, Sweden) was placed under local anesthesia. A stereotactic CT scan was performed, and the image data were transferred to the planning workstation (Framelink 5.0, Medtronic SNT, Louisville, CO). The previously acquired MRI sequences and the DTI FT rendition of the slMFB were co-registered with the stereotactic CT scan and the frame coordinates were extracted.

The first 9 patients were operated with a NexFrame head mounted and navigated stereotactic system (Medtronic, USA). In these cases, 5 fiducial screws were placed, and CT scanning performed. All other patients were implanted using a Leksell G-Frame (Elekta, Sweden). The bilateral DBS electrode implantation was performed under local anesthesia with the patient in a semi-sitting position. Bi-coronal burr-holes were placed sequentially and the burr-hole was sealed with fibrin glue after introduction of the test electrodes in order to prevent CSF loss during surgery. Patients were video-recorded during stimulation.

### 2.6 Microelectrode recording (MER)

**Fig. 4:**
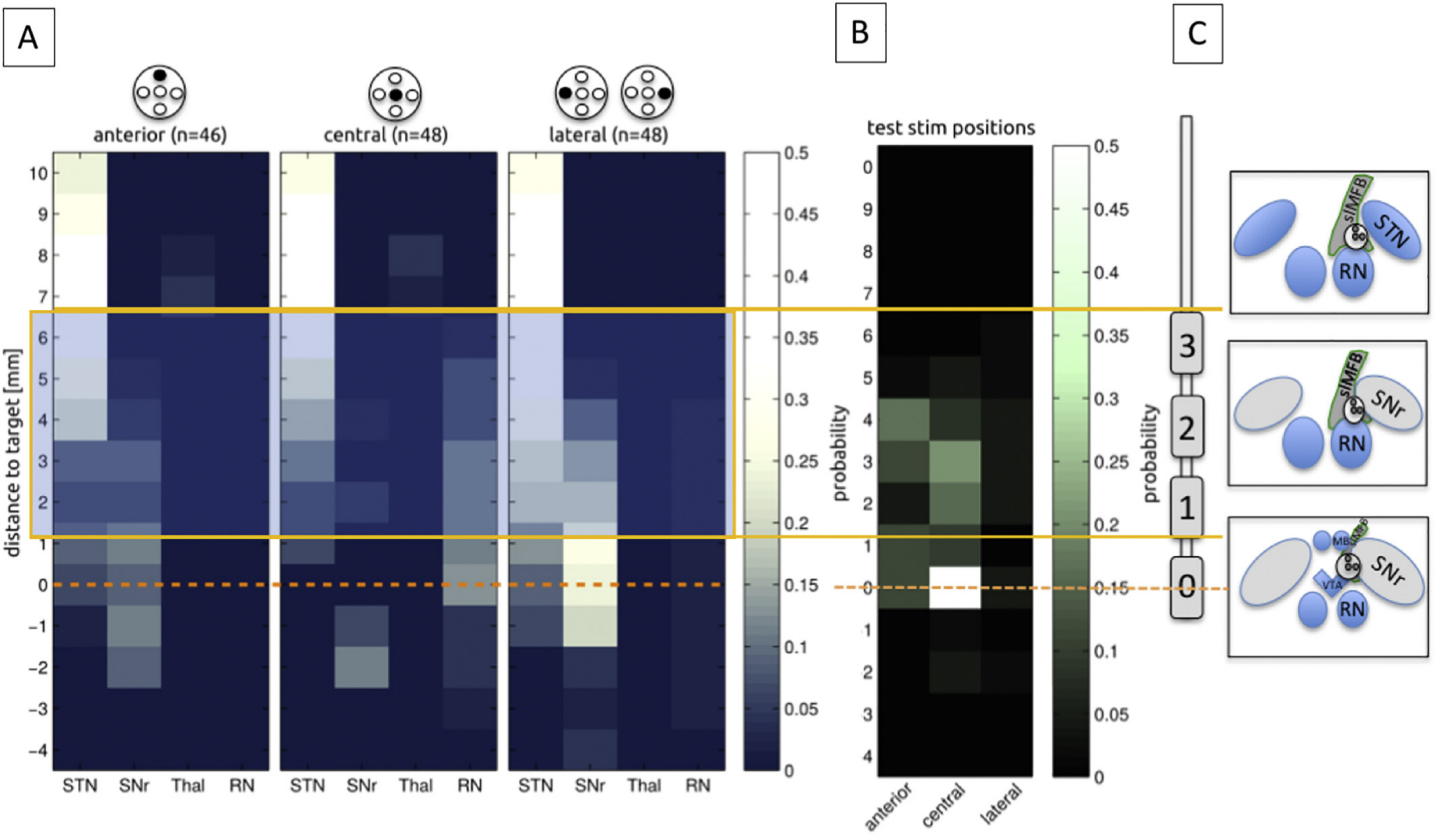
Electrophysiological synoptical graph. A, 144 trajectories and the differentiation in likelihood of occurrence of nuclear structures (STN = subthalamic nucleus; SNr = subtstantia nigra, Thal = thalamus; RN = red nucleus). B, occurrence of test stimulation with respect to the target region; C, typical DBS electrode position and relation to stimulation sites are given. Stimulation was typically performed on contact 1 (anodal) and 2,3 (cathodal). Orange lines indicate overlap with effective stimulation (1.5–6.5 mm above target).

In principle MER in this target region was used to define the white matter corridor between red nucleus (RN) medially and posteriorly, and the STN/SNr-complex, laterally (cf. Fig. 4c). The target region itself is silent in MER besides the inferior-most part of the trajectory which occasionally shows increased activity (interpreted as entry to VTA). Simultaneous recordings from central (c), anterior (a) and lateral (l) trajectories (2 mm spaced apart) were performed starting 10 mm above the target region and extending 4 mm below. For MER a Leadpoint® 8 channel system (Medtronic, USA) with FHC Micro-Macro – Electrodes (MME, FHC, Bowdoin, USA) with a telescopic design and – if expanded – 10 mm distance between micro- and macro-tip inserted via an FHC microdrive (FHC, Bowdoin, USA) were used. On the distinct electrophysiological tracts (a/c/l) the surrounding grey matter structures (red nucleus = RN, subthalamic nucleus = STN and substantia nigra = SNr, Thal = Thalamus) were identified qualitatively recognized based on their specific firing pattern and marked in incremental steps of one millimeter. On the group level the likelihood of occurrence of a certain structure was evaluated with respect to the most inferior point of targeting (inferior border of the slMFB according to tractography) that was defined as target point (“0”). Further electrophysiological evaluation of distinct firing patterns or oscillations in the target region (VTA) were not performed for this study.

### 2.7 Macrostimulation

**Fig. 5:**
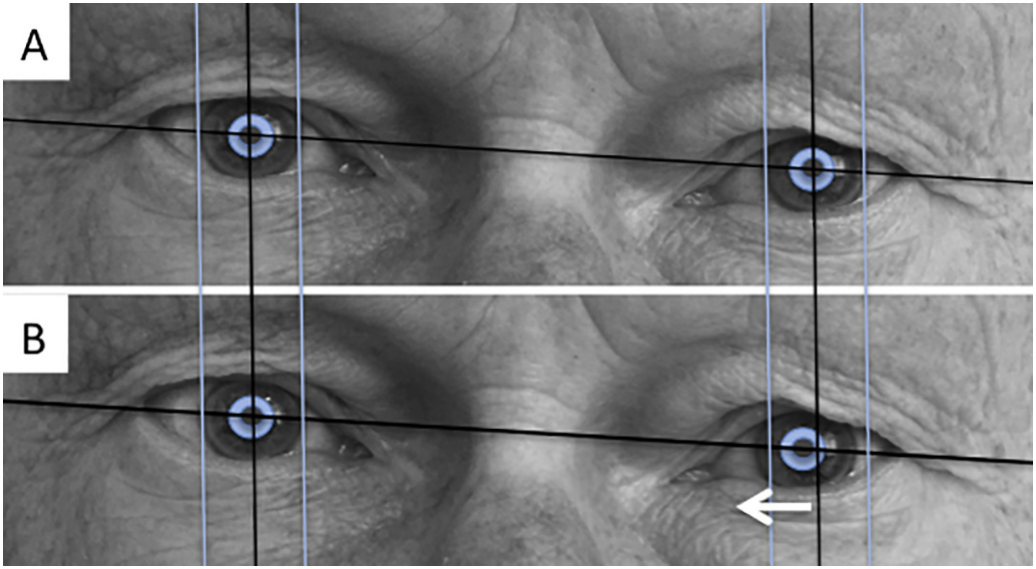
Left oculomotor nerve activation (B) as seen on the deepest stimulation contact and a stimulation current of 1.5 mA (milli-ampere).

Stimulation with the macro-tip of the FHC-Electrode (FHC, Bowdoin, USA) was typically performed on the trajectory (central, anterior, lateral; always clearly within the slMFB) and in a position that showed the *least cellular electro-physiological activity* (MER) above the target region (inferior border of the slMFB). This position was typically found in the middle of the bundle (cf. Figure). Macrostimulation was performed on the fully awake patient fulfilling two purposes: i) to look for acute stimulation effects (“appetitive motivational response”, for details cf. discussion), ii) look for physiological biomarkers that identify the implantation site and guide position of electrode implantation (typically heart rate increase; oculomotor activation). Intra-operative stimulation settings were typically constant stimulation with 1–3 mA, 130 Hz, 60us for several (5–10) minutes. If oculomotor activity was seen in the middle of the bundle at a threshold <3 mA(test-stimulation for heartrate and appetitive motivation in the middle of the bundle) a different trajectory was chosen, still within the slMFB. Depth of final implantation was determined by looking for oculomotor activation at 1.5 mA at the inferior most point of the trajectory (patient reporting double-vision, cf. Fig. 5). Thresholds <1.5 mA led to withdrawal and more superficial positioning of the electrode after repeated testing. Likewise, a threshold >1.5 mA led to deeper (more inferior) testing and final implantation.

### 2.8 DBS electrode and IPG implantation

DBS electrodes (model 3389, Medtronic, USA) were implanted on the chosen trajectory with the deepest point of implantation as described above (oculomotor effect at 1.5 mA). This electrode has 4 contacts (named 0–3) of 1.5 mm length each (1.27 mm diameter) and an interspacing of 0.5 mm. Electrodes were secured in the burr hole cap (StimLock, Medtronic, USA) and final position was checked with lateral fluoroscopy). In a second stage on the same day the patients were placed under general anesthesia and an IPG was placed subcutaneously either in the sub-clavicular or abdominal region. Post-operative helical CT on the day of surgery was performed as described above.

### 2.9 Postoperative titration of stimulation

As shown in Fig. 4, our goal was to locate the tip of the DBS electrode close to the oculomotor response of a patients, indicating lateral VTA (cf. Fig. 1). In order to stimulate the slMFB center a bipolar stimulation was used, typically the second deepest contact (contact 1) was set to anodal (positive) and contacts above (contacts 2 and 3) to cathodal (negative) stimulation (bipolar setting). The amplitude was then increased in order to just not show non-habituating oculomotor effects (double vision or blurred vision, target current typically 2.5 mA).

### 2.10 Evaluation of effectively stimulated contacts (EC)

In order to record the position of effective contacts (EC, a contact that is used for cathodal stimulation) in slMFB DBS we determined their spatial distribution (based on CT fused to the MR-planning) 1. in the mid-commissural point (MCP) coordinate system, 2. with respect to their individual anatomical environment (therapeutic triangle = TT).

### 2.11 Effective contact evaluation with MCP

**Fig. 6:**
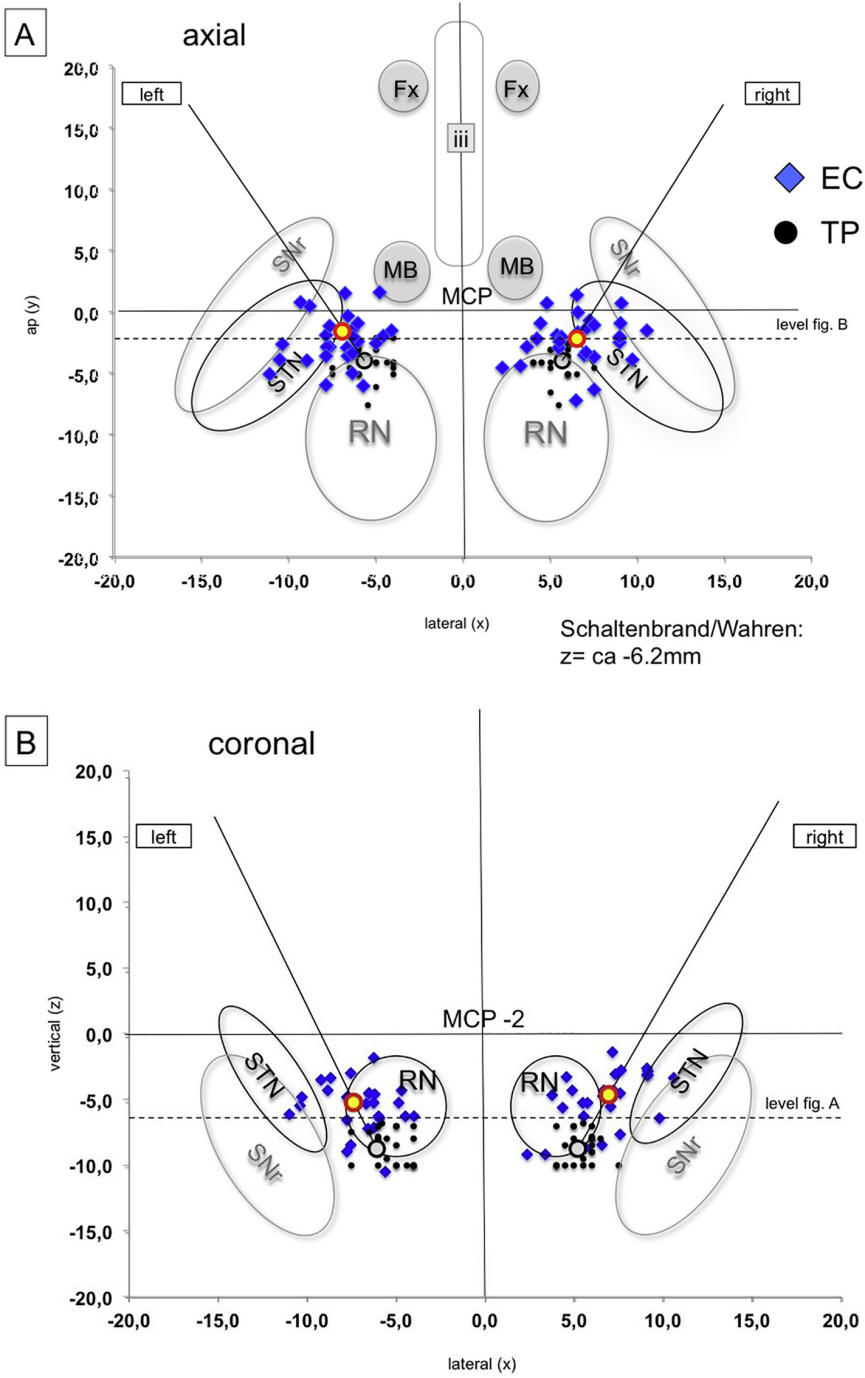

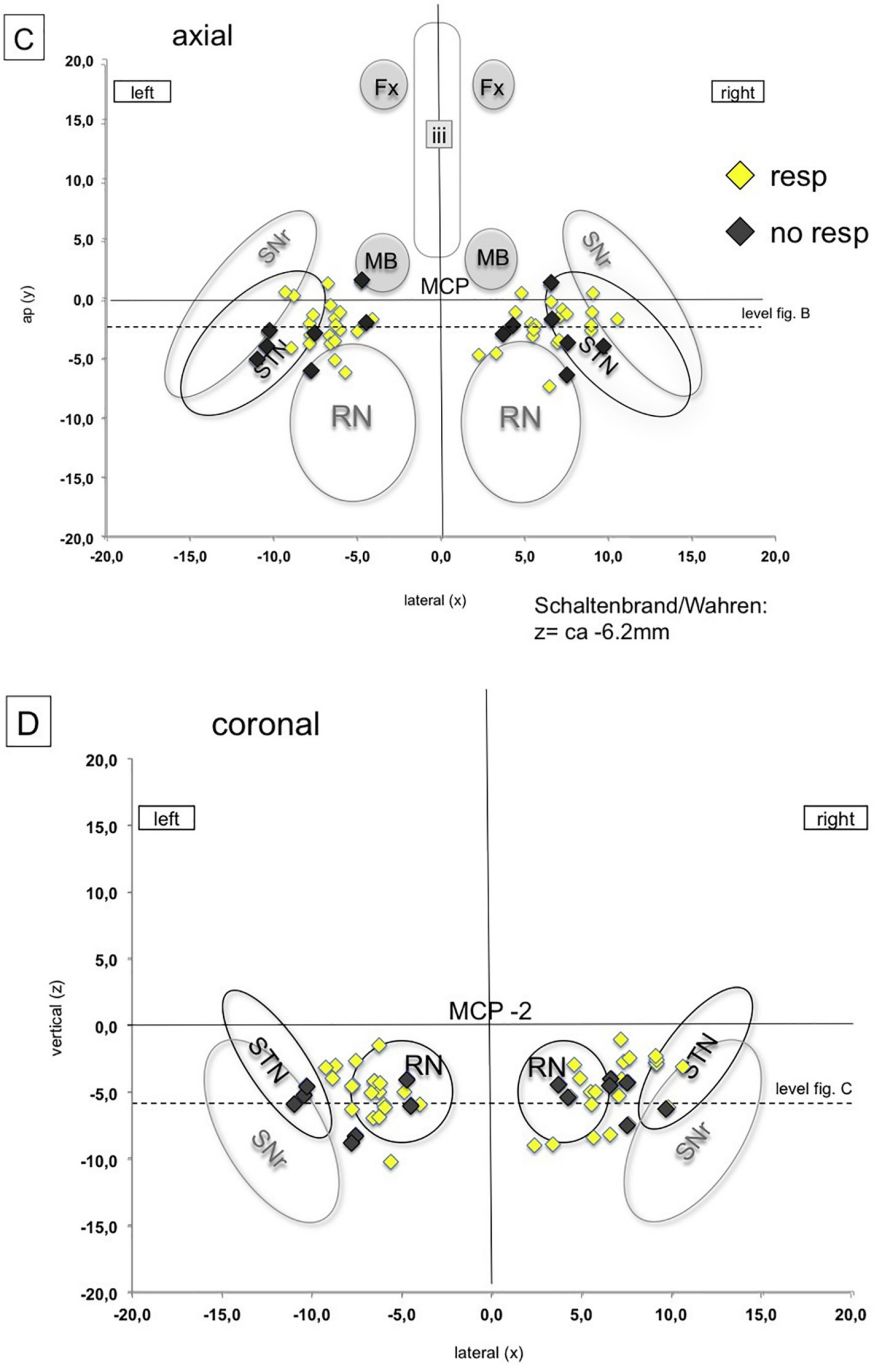
Representation of effective electrode contact (EC) positions in idealized atlas slices (coronal and axial) of the Schaltenbrand and Wahren atlas (Schaltenbrand & Hassler, 1977). Left Panel (A, axial; B, coronal): All EC = blue diamonds, target points (TP) = black dots (projected into the slide in A but in reality, more inferior below the axial plane, cf. B). The mean stimulation point is situated in the corridor between red nucleus and STN/SNr complex. Right panel (C, Axial; D, coronal): Same as A, B but responders yellow, non-responders grey.

Postoperative helical CT data were fused with preoperative MRI and planning data to determine MCP-coordinates of the individually stimulated contact. DBS electrode tips were identified in the planning system (Framelink 5.0, Medtronic SNT, USA) in orthogonal views reconstructed parallel to the electrode (thus a view in direction of the electrode's length axis). Based on the geometry of the 3389 electrodes, the center of an individual EC was determined, and its MCP-coordinate was recorded (cf. Table 3 MCP coordinates; cf. Fig. 6 a–d). Since the typical chronic stimulation was performed in a bipolar fashion with two contacts cathodal (=negative in manufacturere's (Medtronic) nomenclature), *we used the dead space between those two electrodes as the assumed center of the stimulation (assuming similar impedance of both)*. For illustration, electrode positions were plotted on standardized planes (axial, coronal) from the Schaltenbrand and Wahren Atltas (Schaltenbrand & Hassler, 1977).

### 2.12 Effective contacts with respect to anatomical environment/therapeutic triangle (TT)

**Fig. 7:**
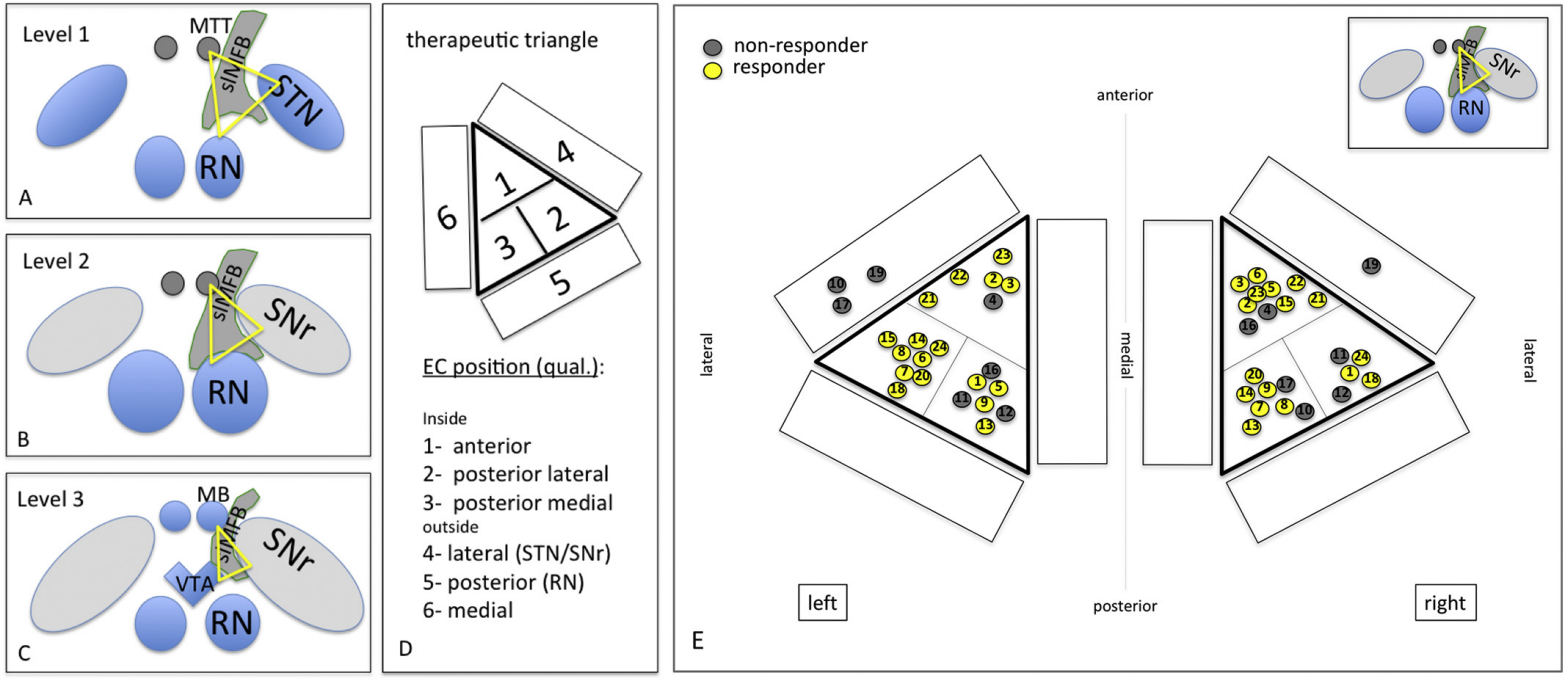
Therapeutic triangle (TT) definition (yellow) between mammillothalamic tracts STN/SNr and red nucleus, respectively. Definition of three stimulation levels for the determination of optimal EC position (A-C). D, sub-parcellation of the TT. E, EC of *responders* (yellow) are clearly located *inside* the TT (projection of EC in level 2, only for visualization purposes). A therapeutic effect is likely due to white matter modulation and not due to an inadvertent stimulation of grey matter structures (nuclei) in the proximity. However, this is not clearly defined by a certain position within the triangle but only by the DTI-FT rendition of the slMFB. For details and statistics see text.

The hypothesis for the optimal stimulation point is that it is located inside the slMFB (as a white matter structure) just lateral and above its exit from the ventral tegmental area. This region is individually determined and targeted with the DTI-tractographic approach (cf. above). However, since the stimulation region is located in a narrow corridor in proximity to the mammillothalamic tract (MTT), the anterior and inferior circumference of the red nucleus (RN) and the anterior and medial border of the subthalamic nucleus (STN)/substantia nigra (SNr) – complex (cf. Fig. 1 topographical sketch) a co-stimulation of these surrounding structures might be possible (cf. Fig. 7,a–c). We defined a therapeutic triangle with respect to the above-mentioned structures. The triangle was then subdivided into three parts (inside: 1 = anterior, 2 = lateral, 3 = medial). The environment outside the triangle was segmented into three additional regions (outside: 4 = lateral, 5 = posterior, 6 = medial). Additionally, we defined three levels of stimulation (1 = above the red nucleus (RN), 2 = at the level of maximal extension of the RN; 3 = below the RN). For details cf. Fig. 7. The postoperative imaging evaluation (fused postoperative CT and preoperative T2W MRI) was then reviewed for the individual patients and EC and graded into a TT scoring for each side separately (example: triangle 3; level 2 = medial inside triangle at the level of the maximal RN extension; cf. Fig. 7A–D).

### 2.13 Statistical evaluation

Analyses were separated for left and right hemisphere. It was not considered that there could be possible interactions between the locations of the respective other electrode and the outcome. Chi^2^-tests were performed comparing response in the different combinations of therapeutic areas. As these analyses are exploratory, there was no adjustment for multiple comparisons. Statistical programming was performed using STATA IC 12.1.

## 3 Results

In total 24 patients (9 female) were implanted in two trials (mean age 47.3 ± 10.5 years; range 29–71 years, FORESEE I & FORESEE II). All patients fulfilled the diagnostic criteria for therapy refractory major depression according to DSM V (for details of inclusion criteria cf. (Schlaepfer et al., 2013)).

### 3.1 Efficacy

Results of the first *n* = 7 patients (FORESEE) have been published (Schlaepfer et al., 2013). Long term results up to 50 months were recently reported form the same cohort (Bewernick et al., 2017) including the neuropsychological outcome (Bewernick et al., 2018) of the same patients. Results from the FORESEE II trial have so far not been published besides preliminary in conference abstracts (Schlaepfer et al., 2016). Results of the first trial were replicated in the second larger study (*n* = 16, n.p,).

### 3.2 *Safety and a*dverse events

Adverse events are fully reported in table 1. Most importantly, there was one intracerebral hemorrhage (FORESEE I), likely related to the micro-electrode recording. This patient has been reported before (Coenen et al., 2013; Schlaepfer et al., 2013). There was one suicide attempt and one hospitalization because of stimulation induced hyperkinesia that ceased after re-programming. Two patients developed infections which made surgical revisions (generator site) necessary. Further adverse events are presented in Table 1. Note the high likelihood of oculomotor symptoms which are idiosyncratic for the target region since the deepest point of the implantation targets the lateral VTA with the oculomotor nerve (CNIII) passing through it. This co-activation of CNIII is actually very helpful in guiding the implantation itself (and also later guiding programming of stimulation).

**Table 1:** Adverse events (for *n* = 24 implantations).

Serious adverse events	Number of patients
Intracranial bleeding (MER)	1
Suicide attempt	1
Partial explantation related to infection (IPG, later re-implantation)	2
Hospitalization because of hyperkinesia	1
Explantation of system on patient's demand^a^ (one in FORESEE I long term f/u, one in FORESEE II)	2
Drug abuse (unbeknown, Methylphenidate) leading to exclusion from study^b^	1
Adverse events	
Transient hemiparesis	1
Dysarthria	1
Hypomania^b^	1
Hypertension	1
Local infection (?) treated with antibiotics	1
*Blurred vision*	*21*
*Doublevision*	*26*
*Strabism*	*2*

a Despite objective antidepressant efficacy.

b Same patient.

**Table 2:** Intraoperative testing and final DBS electrode positions in n = 24 implantations for MDD. More than one third (37.5%) of DBS electrodes were placed in other then the planned (central) trajectory due to the results of MER and intraoperative test stimulation.

DBS electrode positions	*n* = 48 total (100%)	*n* = 30 central (62.5%)	*n* = 10 anterior (20.8%)	*n* = 8 lateral (16.7%)
Occurence of appetitive motivational response (per patient)	n = 24 total (100%)	*n* = 19 bilateral (79%)	n = 4 unilateral (17%)	n = 1 none (4%)
Occurence of increased heart rate (per patient)	*n* = 18 (85.7%)	*n* = 11 bilateral (52.4%)	n = 4 unilateral (19%)	n = 3 none (of which n = 2 with beta blocking agent)
			n = 3 unilateral recorded, only (14%)	*n* = 3 not recorded)
heart frequency increase [bpm]	7.9 (mean)	±5.8 (StdD)		
Occulomotor nerve activation at lowest tested point (per trajectory) cf. Fig. 5	38 total (79,2%)	*n* = 29 inferior border of MFB (60.4%)	*n* = 9 center of MFB (in z-direction) (18.8%)	n = 10 missing data (20.8%)
Occulomotor threshold [mA] ± SD [mA]		1.7 ± 0.9	2.6 ± 1.3

**Table 3:** Planning coordinates/angles and coordinates of effective contacts (in MCP reference system).

Targeting (inferior border of slMFB, DTI FT – assisted targeting)									
	Right			Left			ACPC [mm]	Angle [°] (sagittal)	Angle [°] (coronal)
	X [mm]	Y [mm]	Z [mm]	X [mm]	Y [mm]	Z [mm]			
Mean	5.4	−4.0	−8.6	−5.4	−4.0	−8.6	25.0	62.8	26.6
Min/max	4.0/7.5	−7.5/−2.0	−10/−6.8	−7.5/−4.0	−7.5 /− 2.0	−10/−6.8	22.5/27.5	49.8/73.7	18.2/35.9
SD	0.9	1.2	1.1	1.0	1.3	1.2	1.3	6.1	3.6
Median	5.5	−4.0	−8.5	−5.5	−4.0	−8.5	25.0	61.4	26.7


Effectively stimulated contacts						
	Right			Left		
	X [mm]	Y [mm]	Z [mm|	X [mm]	Y [mm]	Z [mm]
Mean	6.6	−2.3	−4.7	−7.1	−2.4	−5.3
Min/max	2.3/10.6	−7.3/1.3	−9.0/−1.1	−11.0/−4.0	−6.1/1.5	−5.0/10.3
SD	2.0	2.0	2.2	1.8	2.0	1.9
Median	6.6	−2.1	−4.3	−6.6	−2.6	−5.0

### 3.3 DTI tractographic depiction of slMFB

DTI-FT was possible in all cases with an adequate quality that allowed implantation. A typical example is shown in Fig. 2, Fig. 3.

### 3.4 Microelectrode recording (MER)

Results of Micro-recording on 144 trajectories are presented in Fig. 4 and additionally in Table 4 (supplementary material). The typical trajectory enters into the therapeutic triangle in a rather narrow corridor between the surround electro-physiologically active structures (STN, SNr, RN). On the distinct trajectories and above the target region these structures are electro-physiologically recognizable (cf. Fig. 4 and Table 4). The target region might sometimes show higher cellular spiking activity reminiscent of the SNr. We suppose that sometimes we here might have picked up lateral VTA dopaminergic neuron activity which might be difficult to distinguish from SNr neurons (Morales & Margolis, 2017). However, detailed electrophysiological differentiations are not the focus of this work.

**Table 4:**
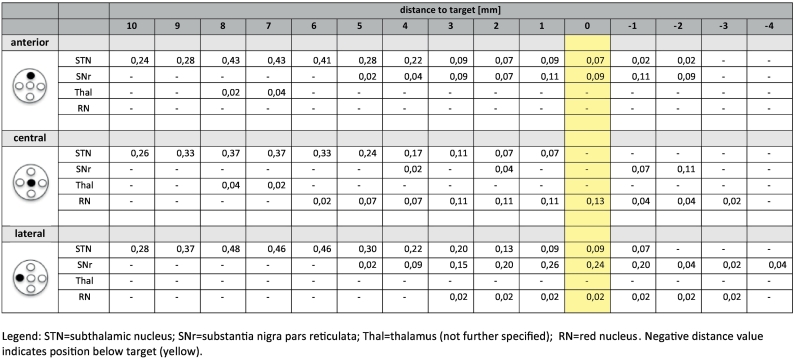
Microelectrode recording (MER) from 24 slMFB DBS procedures (*n* = 142 trajectories). Since the slMFB is a fiber pathway it is expected that it qualifies through a mere “electrophysiological silence “, while the nuclear environment (STN, SNr, Thal and RN) can clearly be identified. Numbers indicate the relative detection frequency of brain regions in bilateral MER recordings (anterior/central/lateral) on the way to the target region. Zero (yellow) represents the planned target point at the inferior most border of the slMFB (as determined with DTI FT) and − 4 is the deepest recorded depth.

Legend: STN = subthalamic nucleus; SNr = substantia nigra pars reticulata; Thal = thalamus (not further specified); RN = red nucleus. Negative distance value indicates position below target (yellow).

### 3.5 Intraoperative macrostimulation

Results of intraoperative macrostimulation are shown in Table 2 and in Fig. 4. Fig. 4 immediately suggest that stimulation on upper electrode contacts (contacts 2 and 3) could potentially lead to inadvertent co-stimulation of structures outside the slMFB (e.g. STN). As further evaluation of potentially co-stimulated regions on distinct trajectories (contacts 1,2,3) with respect to MER we estimated the probability of such a co-stimulation of the STN as the most likely other candidate structure for an antidepressant effect (Coenen et al., 2016; Mallet et al., 2008). For contact 2 (3–4.5 mm above target) the probability of STN stimulation occurring is maximal 0.22 for the anterior (indicating a probability of detecting STN with MER in an anterior trajectory regarding all measured trajectories, cf. Table 4), 0.17 for the central and 0.22 for the lateral trajectories, respectively. Multiplied with the statistical distribution of implantations on a given trajectory (cf. Table 2), there is a probability of 18.2% to co-stimulate the STN on contact 2. Regarding contact 3 (5–6.5 mm above target) there was a probability of 28.6% of such a co-stimulation. In this evaluation the overall probability of co-stimulating the STN (in its most medial and inferior part) is up to 46.8%. However, since we intraoperatively chose the individual MER path that shows the least activity above the target region, we are confident, that realistically the incidence of co-simulation will be much lower than this simulation (see Discussion).

### 3.6 Effective contacts

#### 3.6.1 MCP-coordinates

Detailed targeting coordinates (inferior border of slMFB according to DTI FT) and coordinates of the effective contacts are shown in Table 3. Note the wide range in X, Y, Z with respect to the effective contacts. We have also plotted these contact coordinates inn idealized axial and coronal slices of the Schaltenbrand and Wahren atlas (cf. Fig. 6). This graphic depiction again shows a very wide range of coordinates, making MCP based targeting impossible and showing the need for tractographic targeting. Fig. 6A, B show, that the EC group around our intended stimulation point (mean, yellow and red sphere). Visual inspection shows lack of pattern of distribution between responders/non-responders.

#### 3.6.2 Therapeutic triangle (TT)

The definition and evaluation of the therapeutic triangle is presented in Fig. 7. Responders are defined as showing a reduction of 50% in MADRS (Montgomery-Åsberg Depression Rating Scale) score during at least 50% of the stimulated time period. Contacts of responders (yellow, non-responders grey) are exclusively situated in the center of the triangle with no contact to the nuclear environment. The count of responding contacts in the TT area 1, 2, and 3 on level 2 in the left hemisphere (13 responder, 1 non-responder) was significantly different from the count in the remaining areas (4 responder, 6 non-responder) (Chi^2^-Test, *p* = .005). This might give a hint, that the left hemisphere could be of particular interest. However, due to the very small sample and multiple comparisons (18 comparisons in total), this result should be interpreted with care.

## 4 Discussion

### 4.1 Efficacy

We here described in-detail our implantation procedure for *n* = 24 patients with major depression in two open label trials FORESEE I (Bewernick et al., 2017; Bewernick et al., 2018; Coenen et al., 2013; Schlaepfer et al., 2013) and FORESEE II. Stimulation on the group level showed clear antidepressant efficacy (response being defined as a 50% reduction in the MADRS). In three small published uncontrolled case series, chronic high frequency stimulation of the slMFB appeared to be efficacious. In our own first pilot series six out of seven patients (85%) were responders between 12 and 33 weeks (four out of seven 57%, remitters) (Schlaepfer et al., 2013). In an independent replication, the Houston group published four patients. Two out of three (67%) going into analysis (one dropout) where remitters at 26 weeks after implantation (Fenoy et al., 2016). Long term results of our first cohort (including one extra patient) showed 75% responders (six out of eight patients) at 52 weeks (four out of eight, 50%, remitters) and then seven out of eight patients improved in an area under curve analysis over 50 months (Bewernick et al., 2017). The Houston group have recently published their results (including patients from their first cohort up to 52 weeks) (Fenoy et al., 2018), and found >70% MADRS improvement in five out of six patients reported. There was recently a single case description which showed an improvement in OCD symptoms and depression in a single patient (Oldani et al., 2017). Overall, we are aware of 15 patients that have been published so far. In this small number, antidepressant efficacy of slMFB-DBS appears to be promising.

### 4.2 Depiction of the slMFB with deterministic fiber tracking and its significance for targeting

Since its first description (Coenen et al., 2009), the use of DTI- based tractography has typically used a volume of interested that was placed just lateral to the ventral tegmental area (Anthofer et al., 2015; Coenen et al., 2009; Coenen et al., 2012; Coenen et al., 2018; Fenoy et al., 2016; Schlaepfer et al., 2013). In that sense, the seed region for the local tractographic approach is the same as the effectively targeted and stimulated region. This makes sense since from this strategic point – as a bottleneck - all segments of the slMFB can be addressed by stimulation (Coenen et al., 2018). We cannot be sure if all these distant projections need to be equally stimulated and a sub-separation with tractographic methods is currently performed but beyond the scope of this work. See limitations for discussion of a lack of depiction of distant projections with a single tensor local tractography approach (below). Stimulation is intended at the center of the fiber tract. The tract is a band-like-Structure that traverses the TT. Inferior border and penetration into the respective part of the triangle is defined by the DTI FT. We would at this moment not deviate from this successful strategy.

### 4.3 Macrostimulation and acute effect

We have previously described that the hallmark of efficacious stimulation in addition to the heart rate modulation might be the “appetitive motivation” response. The incidence of this finding in our cohort is presented in Table 2. Typically, this behavior is seen bilaterally but dominantly on one side. The response is a very patient specific and we have not seen such a response in any other stimulation target (especially STN) that we routinely approach in DBS cases for movement disorders. Appetitive motivation describes a goal directed behavior. The patient becomes somewhat more alert (albeit not overactive). There is an exploration of his environment with the eyes. In first cases with a head mounted frame, this response lead to a turn of the head towards the interviewer (not possible with a stereotactic frame). Upon interrogation the patient starts to show interest, which he/she did not show before (e.g. “I would like to go on a vacation” or “I would like to read a book”) We have interpreted this response as positive with respect to electrode position. This response was typically seen when stimulating the center of the slMFB with amplitudes close to oculomotor activation. We have typically seen very similar effects during postoperative initiation of the chronically implanted DBS electrodes and think that this effect is idiosyncratic for the slMFB modulation. We have in previous publications discussed a similarity with the SEEKING response that was described by Panksepp in rodents (Coenen et al., 2011).

### 4.4 A standardized implantation procedure

We were able to create a rather standardized implantation procedure owing to 1. a clear anatomical and reproducible description of the target structure with DTI, 2. reproducible MER that excludes presence of a nuclear and highly active structure in the target region, 3. autonomic side effects (transient heart rate increase) indicating proximity to the target structure, 4. acute effects which are typically lateralized and indicate an express a motivational response under stimulation and finally 5. an oculomotor response (cranial nerve III activation cf. Fig. 5) guiding the implantation depth. In this respect, the implantation procedure includes informative features that allow the neurosurgeon to understand the achieved position and to reverify his implantation during the mere procedure. To this point awake slMFB DBS could be regarded advantageous when compared to other targets that do not present such features (Hamani et al., 2009). In many aspects, typical slMFB DBS implantation is comparable to movement disorder surgery although it has to be performed under tractographic assistance.

### 4.5 Differentiating responders and non-responders

We are certain that a particular brain region must be stimulated in order to elicit response in a patient. To this point and purely based on the analysis of effective contacts performed here (either by MCP reference or TT) we are not able to separate an area that needs to be stimulated in order to differentiate responders from non-responders. On the basis of the TT analysis, responding contacts are typically located inside the triangle. However, we cannot explain, why some non-responder contacts are also situated inside the TT. Moreover, we are not able to explain, why certain responders are situated more medial or lateral, anteriorly or posteriorly inside the triangle. Very likely this is a result of the variability of the slMFB which is targeted based on DTI FT. In this respect, we cannot answer the question if an electrode would be sufficiently placed if it were positioned “anywhere” inside the triangle. At this moment we have to conclude that this surgery should be performed under tractographic assistance since with the use of this technology we and others achieve a high efficacy.

In the first cohort, we detected one left sided hemorrhage (with clinical sequelae – hemiparesis - that resolved within ours) and later argued that the slMFB was unilaterally destroyed in this case with the consequence of a lack of therapeutic efficacy (Coenen et al., 2013; Schlaepfer et al., 2013). While this might be true for this one case, it does not explain, why some patients fail to respond when the target region appears to have been perfectly hit.

### 4.6 Effective contacts: white matter or grey matter stimulation?

We have speculated before, that slMFB-DBS addresses subcortical and cortical reward associated pathways – white matter, the slMFB - by activating descending and ascending fibers towards and from the VTA. We have previously speculated about the mechanism. It is likely that we ortho- and antidromically activate fibers that descend into the VTA and originate in the orbitofrontal and prefrontal cortex (Brodman areae 8,9,10, 11, 11 m) (Coenen et al., 2018). In this concept, the frontal projection is functionally disconnected and cannot communicate with the VTA which is counteracted by modulating the slMFB with HF DBS.

A purely statistical comparative analysis of MER results together with typical electrode positions suggests that the medial and inferior STN might be co-stimulated with an up to 46.8% likelihood. This is a result of a statistical analysis combining the likelihood of the STN in a given trajectory and position with the likelihood of implanting such a trajectory. However, we typically selected the trajectory for implantation of the DBS electrode that showed the least MER activity (see above) and thus likely that our purely statistical estimation of co-stimulation might be too high. Furthermore, a co-stimulation of the STN becomes unlikely in the light of the results of our postoperative imaging and therapeutic triangle analysis (cf. Fig. 7) which clearly lean towards a position of EC inside the TT without contact to surrounding structures (nuclei). The TT certainly is a narrow anatomical bottleneck and we assume that with the expansion of an electric field it is likely that surrounding structures will be co-stimulated to a certain degree. However, the main effective current will spread in the 2 mm proximity (Pilitsis et al., 2008) to the effective contact, VTA simulations notoriously overestimate the size of the true electric field since they typically are based on a homogeneous isotropic environment and other simplifying assumptions (Gunalan et al., 2018) and are not optimal for pure white matter DBS. In the case of slMFB DBS stimulation occurs (if the electrode is optimally placed) inside a white matter tract. During extracellular stimulation of the CNS, these axonal elements represent the most excitable components of neurons surrounding the electrode. A stimulation effect will likely occur at a lower threshold than with grey matter. With respect to current diffusion, Tuch et al. have looked in their modeling approach into the effect of white matter conductivity and found a current spread predominantly in the longitudinal direction of the fiber bundles (Tuch et al., 2001). In our case this would be parallel to the slMFB fibers. Furthermore, white matter will likely prevent lateral current spread due to the high anisotropy of the fiber bundles (McIntyre et al., 2004). However, if an electrode is placed inside or at the border to a grey matter target, predominant diffusion of current into the direction of grey matter would be expected. In the line of this discussion one would expect the current to spread predominantly along the slMFB fibers (especially since bipolar stimulation is used). The TT analysis itself does also not support the hypothesis that a co-stimulation is responsible for the anti-depressant effect (one would expect effective contacts to be grouped more lateral towards the STN). Moreover, the acute effect of stimulation - which we have described as “appetitive motivation response” (see above) is -very typical for the slMFB and is not seen in stimulation of any of the surrounding grey matter structures (STN, SNr etc.). Dyskinesias are not a typical side effect of our stimulation (Tables 1, 1 case of hyperkinesia due to suboptimal programming that resolved after stimulation adjustment, programing to a further distal – inferior - contact) suggesting that at least the sensory-motor part of the STN is not reached. Furthermore, we have seen only one case of hypomania (cf. Table 1, abuse of Methylphenidate) and it was clearly not stimulation induced. Tributaries of the medial (limbic) STN to the slMFB (Coenen et al., 2018) have been mentioned as the causative agent of hypomania in STN DBS in Parkinson's disease (Coenen et al., 2009) and we have proposed earlier that this (in Parkinson's disease pathological) activation of the reward system might account for a proposed antidepressant efficacy in major depression (Coenen et al., 2011). Furthermore, it is likely that part of the anti-obsessive compulsive effect (anti OCD and in part antidepressant effects) which have been reported for medial STN DBS (Mallet et al., 2008) is attributable to a diffusion of current into the same tributaries. A similar reasoning was recently applied in an own pilot series of slMFB DBS in OCD (Coenen et al., 2016). To this end, it appears to be not important if there is an unwanted direct co-stimulation of the medial and inferior STN or an intended stimulation of its tributaries to the slMFB (Coenen et al., 2018) (cf. Fig. 1). Nevertheless, inadvertent co-stimulation of surrounding structures should be kept in mind as one of the potential mechanisms of the antidepressant effect of slMFB-DBS.

Other circumstances might come into play for a lack of antidepressant efficacy like patient selection and phenotyping. Separation of responders and non-responders, however, is not the focus of this work but is the topic of our ongoing research.

### 4.7 Limitations

Neuronavigated vs. stereotactic frames: The first cohort of patients was operated with a head mounted frame (NexFrame, Medtronic, USA). We changed the procedure after these cases because a lack of confidence emerged in the accuracy of the neuro-navigated frame in a clinical case (not in this series). The procedure was adapted to a stereotactic frame. Besides some restrictions in the possible head movement with a stereotactic frame there were no gross differences in the procedure. The placement of the DBS electrodes did not show any obvious differences in placement accuracy between the two frame types.

Microelectrode recording: In this work we present steps that lead to a safe and efficacious implantation of DBS electrodes in the slMFB. Therefore, we only qualitatively evaluated MER signals. A further evaluation of the electrophysiological characteristics - especially the SNR and VTA in major depression – is interesting and part of our future work but beyond the scope of this work.

*The use of deterministic tractography* (DT) for targeting of the slMFB must be discussed, especially when looking at the many connections this structure makes cortically and subcortically. While on the group level DT might be able to show most of these distant projections (Coenen et al., 2012), on the single subject level this is typically not possible (Anthofer et al., 2015; Hana et al., 2015) even when using advanced software solutions (Anthofer et al., 2015; Coenen et al., 2018). Advanced tracking methodology including multi-shell imaging and holistic tractography approaches are able to show the complete extension of the slMFB (Coenen et al., 2018). However, these algorithms to this point are not part of the advanced stereotactic and tractography planning tools that are commercially available and CE- or FDA-marked for such a purpose. Other groups who now also target for fiber tracts for other target regions have started to compare idealized tractographic templates (derived from more advanced tracking methods) to draw conclusions out of their DT results (Riva-Posse et al., 2014; Riva-Posse et al., 2017). When it comes to surgical planning, most importantly the target region has to be displayed with acceptable accuracy. In a recent study we showed, that local tractography approaches (like the deterministic local “one tensor”- approach) lead to very similar results as more advanced tracking methods (probabilistic or global tracking) when looking at the slMFB trunk as the identified target region, directly (Coenen et al., 2018). For the time being it thus might be acceptable to use local approaches when strictly regarding the limitations of this technology.

Separation of the effectively stimulated brain tissue that helps to differentiate responders from non-responders warrants a further analysis. Detailed electrode positions and elaborated field simulation studies of the electromagnetic field (Gunalan et al., 2018; McIntyre et al., 2004; Noecker et al., 2018) should be applied in order to understand the effectively stimulated brain regions and the structures inside that are involved. These analyses, however, are beyond the scope of this work and a focus of future publications.

## 5 Conclusions

The medial forebrain bundle is an important structure of reward and motivation. The slMFB emerges as a potential region for the treatment of major depression (and other psychiatric diseases) with DBS. So far it has only been successfully researched in open label clinical case series with results published from no >15 patients. The detailed surgical procedure of slMFB DBS implantation has not been described before. Stimulation produces its efficacy likely through direct white-matter modulation of slMFB fibers and not via a co-stimulation of the grey matter environment, although we cannot completely rule out such a possibility. The implantation procedure for slMFB DBS comprises a standardized protocol combining tractographic imaging based on DTI FT, targeting and electrophysiological evaluation of the target region. Our evaluation of MCP-coordinates shows a very wide interindividual range, making MCP-based targeting impossible. Therapeutically effective contacts need to be located in the therapeutic triangle but their individual detailed position inside the triangle is determined by individual DTI FT of the slMFB.

Some informed features (MER exclusion of an actively spiking functional environment, heart rate increase, acute stimulation effects, oculomotor activation) help to readily identify the target region on the operation table. These aspects allow the neurosurgeon to directly control and optimize his part of the multidisciplinary approach to slMFB DBS in MDD. To this end, slMFB DBS surgery is in many aspects comparable to typical movement disorder surgery and form the viewpoint of implantation may be more advantageous then other target regions for MDD that lack such features. However, this advantage does not automatically imply a clinical superiority of slMFB in treating MDD and this latter aspect is currently being researched. In our view, slMFB DBS should only be performed under tractographic assistance. We have here described in detail our surgical experience in 24 cases of slMFB DBS in MDD. This description might in the future help other groups to successfully and safely implement the surgical approach to the slMFB for the treatment of psychiatric disorders.

## Conflict of interest statement

This work is based on two Investigator Initiated Trials (FORESEE I & FORESEE II) that were partly funded (mainly generators) by Medtronic (USA).
